# Life history enlightened therapies: cell cycle mapping to identify molecular targets to prevent hepatocellular carcinoma

**DOI:** 10.1093/emph/eoag002

**Published:** 2026-01-14

**Authors:** Anuraag Bukkuri, Janet McLaughlin, Andrew W Duncan, Wayne Stallaert

**Affiliations:** Department of Computational and Systems Biology, Hillman Cancer Center, Center for Evolutionary Biology and Medicine, University of Pittsburgh, Pittsburgh, PA, USA; Department of Mathematics, City St George’s, University of London, London, UK; Department of Computational and Systems Biology, Hillman Cancer Center, Center for Evolutionary Biology and Medicine, University of Pittsburgh, Pittsburgh, PA, USA; Department of Pathology, McGowan Institute for Regenerative Medicine, Pittsburgh Liver Research Center, University of Pittsburgh School of Medicine, University of Pittsburgh, Pittsburgh, PA, USA; Department of Bioengineering, School of Engineering, University of Pittsburgh, Pittsburgh, PA, USA; Department of Computational and Systems Biology, Hillman Cancer Center, Center for Evolutionary Biology and Medicine, University of Pittsburgh, Pittsburgh, PA, USA

**Keywords:** life history enlightened therapies, endocycling, cell state transitions, hepatocellular carcinoma, multiplexed immunofluorescence, cell cycle mapping

## Abstract

**Background and objectives:**

Life history enlightened therapies (LHETs) were originally developed in cancer to combat therapeutic resistance by targeting pathological cell state transitions that enable evolutionary rescue and adaptation to therapy. In this paper, we expand the scope of LHETs to cancer prevention, namely, metabolic dysfunction-associated steatohepatitis (MASH)-induced hepatocellular carcinoma (HCC) initiation. We focus on endocycling, a process wherein cells undergo whole-genome duplication via mitotic skipping, as a keystone life history transition that promotes HCC initiation.

**Methodology:**

A key obstacle to LHET translation is the lack of systematic methods that capture cellular life histories and their molecular drivers. To address this gap, we introduce cell cycle mapping, a technique that integrates *in situ* multiplexed immunofluorescence imaging with manifold learning to visualize the life history of proliferating cells and proteins involved in transitions to pathological cell states.

**Results:**

Mathematical modeling demonstrated how endocycling can create an environment that promotes HCC initiation and revealed the potential for endocycle-targeting therapies to prevent HCC. Using cell cycle mapping on human MASH liver tissues, we identified the molecular factors that drive pathological endocycling (Wee1, CDK2, and RAIDD), providing new therapeutic targets for pre-clinical investigation.

**Conclusions and implications:**

This application illustrates how cell cycle mapping can uncover key proteins that drive disease-associated cell state transitions and broaden the scope of LHETs from therapy resistance to cancer prevention. More broadly, our study establishes a generalizable pipeline for inferring the life history of cells in diseased tissues and potential interventions for disease management.

## BACKGROUND AND OBJECTIVES

Life history theory is an evolutionary framework [1, 2] that seeks to understand how organisms allocate limited resources toward biological processes such as growth, survival, and reproduction, and the implications of these trade-offs on organismal dynamics. Life history theory plays a crucial role in informing management strategies for species conservation and population recovery. It is used to estimate extinction risk through population viability analysis, identify the most vulnerable life cycle stages for targeted intervention, and design recovery plans that support long-term population persistence. For instance, consider the case of the loggerhead sea turtle. A threatened species in the late 20th century, early recovery efforts focused on protecting nests and conserving the egg state due to ease of implementation and accessibility of this life cycle stage. However, life history analysis demonstrated that this approach was the least effective method for protection efforts and instead suggested that adult mortality had the greatest impact on population growth [3]. These results inspired efforts focused on fisheries bycatch that proved to be effective and paved the path for the World Trade Organization Shrimp-Turtle case in 1994, which mandated the use of turtle excluder devices on shrimp trawls [4]. This case study exemplifies how life history theory offers a powerful framework for understanding complex biological systems and guiding effective management interventions.

In prior work, we applied such ideas to design life history enlightened therapies (LHETs) to combat cancer therapeutic resistance [5–8]. As the “evil twin” of conservation efforts, our goal here is to harness life history theory to promote eradication of harmful cells. In multiple cancer types, chemotherapy can divert cancer cells that have successfully completed DNA replication along an alternative trajectory that bypasses mitosis to induce whole-genome duplication (WGD)—a process called endocycling^9–13.^ This pathological state provides a refuge for the cells from therapy, allowing for evolutionary rescue and subsequent adaptation to the stressor. Recognizing this life history and its contribution to drug resistance, we designed LHETs that combined chemotherapy with a drug that targeted this therapy-induced endocycling. This evolutionary double bind significantly reduced tumor burden, leading to an approved clinical trial (NCT05574712) for treatment of regional prostate cancer and promising preclinical results in a variety of other cancers [8]. However, a systematic way to identify appropriate drugs to target these pathological cell state transitions is lacking, as it is difficult to capture and measure life history of individual cells. Moreover, live cell experimental models that accurately capture human disease states are rare, and life histories often need to be inferred from tissue samples taken directly from patients and subsequently chemically fixed or frozen. In prior work, we introduced a novel method called cell cycle mapping to visualize the life history of proliferating cells and the mechanisms that govern their transitions through the canonical phases of the cell cycle [9, 10]. Notably, in response to the chemotherapeutic agent etoposide, this approach revealed the point at which cultured retinal epithelial cells diverted from the canonical cell cycle to the endocycle trajectory leading to WGD [10]. In this paper, we apply this technique to interrogate the life history of cells in their native diseased context to identify targetable proteins that drive pathological endocycling in a rigorous, data-driven manner. Notably, this is the first paper that performs cell cycle mapping to interrogate cellular life histories from fixed tissue samples.

We previously used LHETs in the context of therapeutic resistance. Here we turn to a related but distinct challenge: cancer initiation in the context of hepatocellular carcinoma (HCC). HCC is the most common type of liver cancer and is a leading cause of cancer deaths worldwide, taking the lives of over 800 000 people each year. The shortage of healthy donor livers for transplantation, coupled with systemic treatments that minimally extend survival have limited outcomes for patients with HCC. This makes HCC prevention all the more important. The fastest growing cause of HCC is metabolic dysfunction-associated steatohepatitis (MASH), a chronic liver disease associated with senescent, inflammatory, and fibrotic (SIF) [11–15]. This trend is particularly apparent in the USA, with its rapidly rising levels of obesity and diabetes. Thus, efforts to stall MASH progression will be particularly fruitful in decreasing HCC incidence.

A key cellular process that occurs with MASH progression is endocycling, in which cells undergo WGD without entering mitosis. Namely, cells replicate their deoxyribonucleic acid (DNA), exit the cell cycle in G_2_, enter a senescent-like state via mitotic skipping, and re-enter the cell cycle in G_1_ without having undergone cell division [16, 17]. Cells generated via such pathological polyploidization exacerbate the SIF phenotype and further MASH progression [18–23]. The contribution of endocycling to tissue remodeling and disease progression has been shown in a variety of contexts. In the liver, endocycling in a *CDK1^Liv−/−^* mouse model was linked to inflammation and fibrosis, leading to liver damage (elevated blood alanine transaminase, bilirubin, and alkaline phosphatase levels at P14) despite a healthy background [23]. Similarly, endocycled tubular cells in the kidney adopted a senescent and profibrotic phenotype, culminating in TGF$\beta$1 expression that further promoted endocycling via YAP1 in a positive feedback loop and drove chronic kidney disease [18–20].

Cell cycle analysis of hepatocytes from genetic and dietary mouse models of MASH and patients with MASH shows a significantly elevated level of *mononuclear*, polyploid hepatocytes, characteristic of endocycling [22]. In this paper, we use qualitative mathematical modeling to demonstrate how drugs that target the pathological endocycle may serve as a promising avenue to prevent MASH progression and ameliorate HCC initiation by reducing both the frequency of cancerous mutations and restoring immune controls on pathological proliferation. We then use cell cycle mapping to visualize the life histories of cells *in situ* and elucidate the molecular factors that divert cells toward endocycling. These results will inform future preclinical studies by providing drug targets that will be validated as a LHET using *in vivo* mouse models that capture the MASH to HCC transition.

## METHODOLOGY

### Mathematical Modeling

To understand how endocycling contributes to MASH-induced HCC initiation and to evaluate the potential of endocycle-targeting therapies for disease prevention, we used mathematical modeling. Namely, we constructed a system of ordinary differential equations to capture the population dynamics of normal (N), endocycle (E), and cancer (C) cells in the liver:


\begin{eqnarray*}&&\frac{dN}{dt}=\alpha \left(1-\mu \left(\boldsymbol{X}\right)\right)N\left(\frac{K-N-E-C}{K}\right)-\delta N-{\varphi}_N(t)N \\ && \quad\qquad + 2{\varphi}_E(t)\\&& \frac{dE}{dt}={\eta \varphi}_N(t)N-{\varphi}_E(t) -\delta E \\&&\frac{dC}{dt} = \beta C\left(\frac{K-N-E-C}{K}\right)-\delta C-\kappa \left(\boldsymbol{X}\right)C\\&&\quad\qquad + \alpha \left(\mu \left(\boldsymbol{X}\right)\right)N\left(\frac{K-N-E-C}{K}\right)\end{eqnarray*}

Here, $\boldsymbol{X}$ is a vector capturing our state variables $\left[N,E,C\right]$, $\alpha$ and $\beta$ capture the intrinsic growth rates of our normal and cancer cells, $\mu \left(\boldsymbol{X}\right)=\frac{0.01}{1+{e}^{-0.4\left(\frac{100E}{E+N+C}-5\right)}}$ is the mutation rate, $K$ is the carrying capacity, $\delta$ is the intrinsic death rate, ${\varphi}_N(t)=\left\{\!\!\!\! \begin{array}{c} 0\ \mathrm{for}\ \mathrm{t}<\mathrm{\psi} \\ \frac{0.02}{1+{e}^{-.06\left(t-125\right)}}\mathrm{for}\ \mathrm{t}\ge \mathrm{\psi} \end{array}\right.$ and ${\varphi}_E(t)=0.01+\frac{0.04}{1+{e}^{.06\left(t-125\right)}}$ are the transition rates from normal to endocycle cells and vice versa, where $\mathrm{\psi}$ represents the induction of chronic stress, and $\kappa \left(\boldsymbol{X}\right)=0.01+\frac{0.15}{1+{e}^{0.4\left(\frac{100E}{E+N+C}-5\right)}}$ captures immune-mediated cancer cell death.

As with all models of reality, our mathematical models make a number of simplifying assumptions. We presume that normal and cancer cells grow in a logistic manner, that cancer cells have a higher intrinsic growth rate ($\beta >\alpha$), and that unidirectional mutations from normal to cancer cells occur. Due to the inflammatory phenotype that accompanies endocycling and the increased genomic content that allows for evolutionary experimentation, we let the mutation rate increase as a function of the frequency of endocycling cells in the tissue [24–28]. For simplicity, we assume that endocycling cells cannot divide to produce further additional endocycling progeny but can undergo reductive mitosis at a low rate to produce two normal cells. We assume that all cells die at the same background rate ($\delta$) and cancer cells are killed by the immune system at a rate $\kappa$. Since endocycling cells inhibit immune infiltration and activation through their SIF profile [24, 25, 29], we let $\kappa$ decrease as a function of the frequency of endocycling cells in the liver. Table 1 lists the baseline parameter values used in our simulations.

**Table 1:** Parameter interpretations and values used in simulations.

Parameter	Interpretation	Value
$\alpha$	Normal cell intrinsic growth rate	0.3/day
$\beta$	Cancer cell intrinsic growth rate	0.45/day
$K$	Carrying capacity	10 000 cells
$\delta$	Intrinsic death rate	0.1/day
$\mu \left(\boldsymbol{X}\right)$	Mutation rate	[0,0.01]/division
${\varphi}_N(t)$	Endocycle rate	[0,0.02]/day
$\eta$	Probability of successful endocycle	1
${\varphi}_E(t)$	Ploidy reduction rate	[0,0.05]/day
$\kappa \left(\boldsymbol{X}\right)$	Immune-mediated cancer cell killing	[0.01,0.16]/day

### Cell cycle mapping

**Figure 1:**
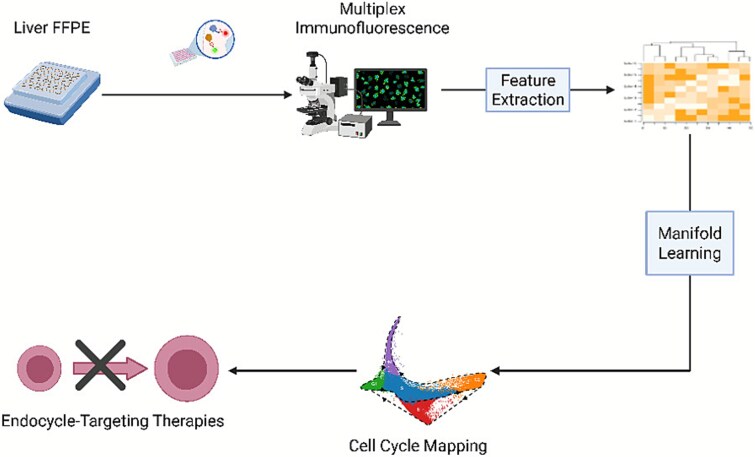
Depiction of *in situ* cell cycle mapping strategy. Formalin-fixed paraffin-embedded tissues from patients with MASH were obtained and stained with several antibodies for key cell cycle regulators. Deep learning enabled segmentation of cells and quantification of protein expression levels. Manifold learning was applied upon this large data matrix to visualize the progression of cells through the cell cycle in a two-dimensional embedding called a cell cycle map. This map can then be investigated to identify key proteins involved in the pathological endocycle and identify drug targets to targeted endocycling and implement LHETs. Created using Biorender.com.

To ascertain the molecular factors that drive endocycling, we performed *in situ* cell cycle mapping [30] (Fig. 1). We obtained a tissue microarray (TMA) from BioIVT (New York, USA) containing liver samples from 26 MASH patients. We performed iterative immunofluorescence [31] (CellDIVE Imager, Leica Biosystems, Nussluch, Germany) to stain and image 17 key cell cycle regulators, including key cell cycle regulators and proteins with a known role in WGD: pRB, AKT, p16, p21, RB, CDK6, Cyclin E2, Cyclin D1, CDK2, Wee1, Cyclin E1, p27, Smad4, CDK1, RAIDD, MDM2, and cMyc. These molecular factors have successfully been used in prior work to map the cell cycle and capture endocycling [9, 10].

Whole cell and nuclear segmentation were performed using Cellpose [32], which enabled subcellular quantification of each of the proteins measured in the nucleus and cytoplasm of every cell across all tissue samples. This allowed us to obtain a detailed, proteomic signature of the cell cycle “state” for each cell in our TMA. These features were z-score normalized to ensure comparability across tissue samples. After filtering out non-proliferative pRB- cells, we performed manifold learning using Potential of Heat-diffusion for Affinity-based Transition Embedding (PHATE) [33]. This non-linear dimensionality reduction algorithm, which is designed for visualizing continuous, branched biological processes as lower-dimensional embeddings, allowed us to generate a 2-day cell cycle map that captures the life history of proliferative cells in their native environment.

To identify discrete cellular subpopulations within the cell cycle state space, we constructed a k-nearest neighbors graph from the standardized feature data. The graph was passed into an unsupervised Leiden clustering algorithm, a robust community detection method, generating cluster assignments that primarily corresponded to the canonical cell cycle phases (G1/S/G2/M) and endocycling. We visualized the PHATE embedding with overlaid color maps representing individual marker intensities. This enabled assessment of how features varied across the manifold and within inferred clusters. Importantly, this interpretable machine learning approach allowed us to identify the molecular factors that target cells toward endocycling.

## RESULTS

**Figure 2:**
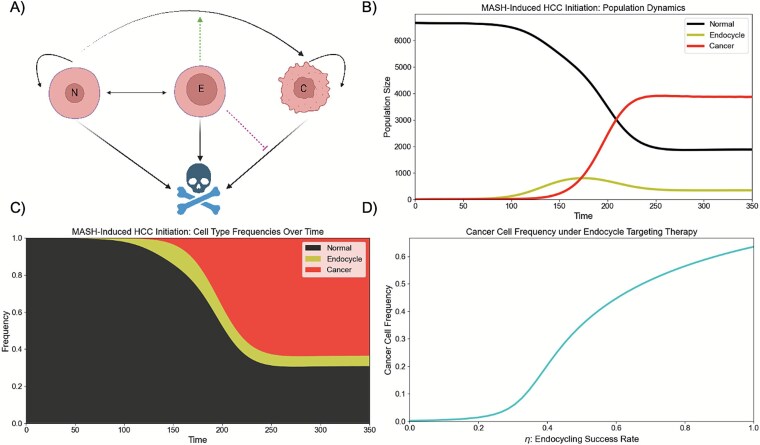
Mathematical modeling of the impact of endocycling on MASH-induced HCC initiation. (A) Structure of the mathematical model: Cells can transition between the normal and endocycle state, cancer cells can mutate to become cancerous, and normal, endocycle, and cancer cells are all subject to death. Endocycling promotes mutations from normal to cancer cells due to its inflammatory phenotype and reduces cancer cell death by inhibiting immune activation and infiltration. Created using Biorender.com. (B & C) population (B) and frequency (C) dynamics of normal, endocycle, and cancer cells: Chronic stress begins at time 50 and promotes endocycling. This then creates a SIF environment that paves the path for cancer invasion. (D) Population dynamics under endocycle-targeting therapy: Blocking pathological endocycling ameliorates this SIF phenotype hampers cancer cell invasion in the tissue.

To understand how endocycling contributes to MASH-induced HCC initiation, we ran simulations of our mathematical model. This model captures the population dynamics of normal, endocycling, and cancer cells in the liver. It allows for proliferation of normal and cancer cells, switching between normal and endocycling cells, mutations from normal into cancerous cells, natural cell death, and immune-mediated cell death for cancer cells. Endocycling-induced tissue remodeling, due to increased inflammation and fibrosis, is assumed to increase mutation rate and decrease immune-mediated cancer cell killing (Fig. 2A). For simplicity, we considered a purely ecological model that focuses on the effects of endocycling on HCC initiation. However, there are several other factors that contribute to the increased evolvability of cancer [34–38], including hypoxia-driven genomic instability [39, 40], reactive oxygen species production [41, 42], and acidosis-induced mutagenesis [43, 44]. Although considering the evolutionary dynamics and reciprocal interactions among normal hepatocytes, endocycling hepatocytes, cancer cells, and the liver microenvironment is outside the scope of the paper, these factors will be critical to consider in future work that connects MASH-induced HCC initiation with subsequent progression and therapy resistance.

We first ran a simulation of MASH-induced HCC initiation in the absence of therapeutic intervention (Fig. 2B,C). We allow the tissue to maintain homeostasis for the first 50-time steps and then induce chronic stress, captured by non-zero rates of endocycling. This eventually paves the path for the proliferation of cancer cells in the tissue by increasing the rates of normal to cancer cell conversion and decreasing the immune controls on the cancer cell population.

The environmental degradation driven by endocycling is reminiscent of problems addressed in restoration ecology. This discipline, with its focus on restoring ecosystems impacted by disturbances such as pollution, habitat loss, and invasive species to their natural states, provides a novel framework for thinking about HCC prevention. Namely, we hypothesize that therapies that specifically target endocycled hepatocytes, the *keystone species* within the diseased liver ecosystem, by blocking transitions from the canonical mitotic cycle to the endocycle will prevent the ecosystem engineering effects of endocycling (SIF), re-establish a healthy liver microenvironment, and inhibit HCC initiation. Similar in flavor to our cancer, corruption, and criminology research program [45–47], this approach adopts a synchronic reflexive perspective rather than a mereological one, focusing on how the complex interplay between microscopic and macroscopic factors gives rise to pathology. To test this hypothesis, we ran the same simulation but prevented successful endocycling to varying degrees (Fig. 2D). As expected, although normal cells still attempt to undergo WGD in response to chronic stress, the more effectively endocycle cells are prevented from forming, the fewer cancer cells are able to invade the tissue.

**Figure 3:**
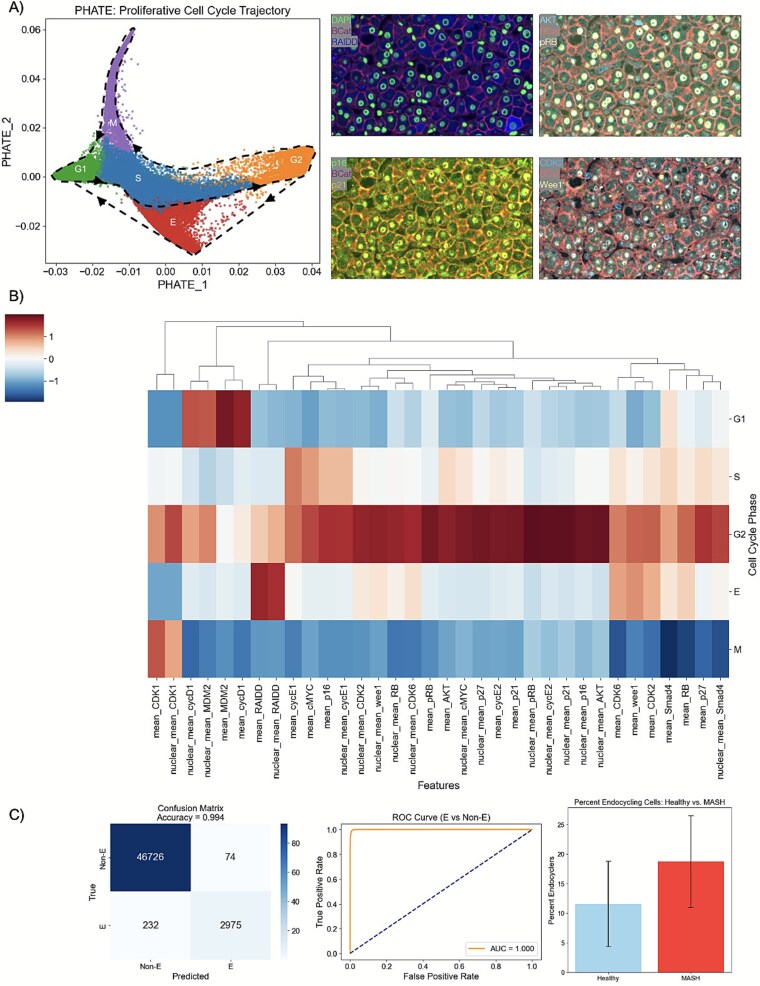
(A) Antibody staining sample images and cell cycle map clustering. Our clustering algorithm identified five distinct clusters, corresponding to G_1_ (green: left), S (blue: center), G_2_ (orange: right), endocycle (red: lower), and mitosis (purple: upper). (B) Heatmap to visualize differential protein expression of all features across all cell cycle phases. C) Our (validated) random forest classifier predicts a higher frequency of endocycling in the livers of patients with steatohepatitis compared to healthy controls.

Given that blocking the endocycle may prevent MASH progression and HCC initiation, the question arises: How do we map a dynamic process such as endocycling within intact, fixed MASH tissues to identify potential drug targets that mediate this pathological cell state transition? To address this question, we turned to our cell cycle mapping approach. Using multiplexed immunofluorescence imaging and deep-learning-based cell segmentation, we obtained single-cell proteomic measurements of 17 key cell cycle effectors in 26 FFPE MASH tissue specimens. Hepatocytes were selectively segmented using β–catenin expression, and non-proliferating cells with low levels of RB phosphorylation were identified and filtered out using a Gaussian mixture model. These data were projected via manifold learning onto a two-dimensional cell cycle map that successfully captured sequential transitions through the phases of the canonical cell cycle (G1-S-G2-M) (Fig. 3A). We then examined the maps to determine key factors driving endocycling in MASH.

Figure 3 shows samples of our antibody imaging and the results from our cell cycle mapping. Our multiplexed immunofluorescence approach enables tissues to be repeatedly stained with several rounds of antibodies, allowing each cell to be measured across all our proteins of interest. Our clustering algorithm identified five clusters, corresponding to cells in G_1_ (green), S (blue), G_2_ (orange), endocycle (red), and mitosis (purple). Cells begin in the green G_1_ cluster before proceeding into the blue S phase, then into the orange G_2_, and up into the purple mitotic phase before re-entering the cell cycle in G_1_. We notice a clear divergence of the endocycle from the canonical cell cycle, wherein cells transition from G_2_ into the red endocycle before re-entering the cell cycle in G_1_ without cell division.

Using hierarchical clustering, we obtained four distinct clusters corresponding to the proliferative cell cycle phases (G1/S/G2/M) by the expression of conserved biomarkers, and a fifth cluster we hypothesized to be endocycling cells. To characterize features specific to the endocycle, we first quantified their expression across cell cycle phases defined by our clustering algorithm. We then trained a random forest classifier to discriminate endocycle from non-endocycle cells, achieving an AUC of 1.000 and an accuracy of 0.994 using a 70/30 train-test split on our cell cycle map data. The top features to distinguish endocycle from non-endocycle cells were RAIDD, pRB, CDK1, Wee1, AKT, p21, CDK6, Cyclin E2, p27, Cyclin E1, CDK2, and p16 (Supplemental Fig. 1). We used this random forest classifier to capture the percent of endocycle cells across our healthy tissue samples and compared the results to the percent of endocycle cells in our MASH tissue samples. As expected, we found a higher frequency of endocycling in patients with steatohepatitis compared to healthy controls (Fig. 3C).

**Figure 4:**
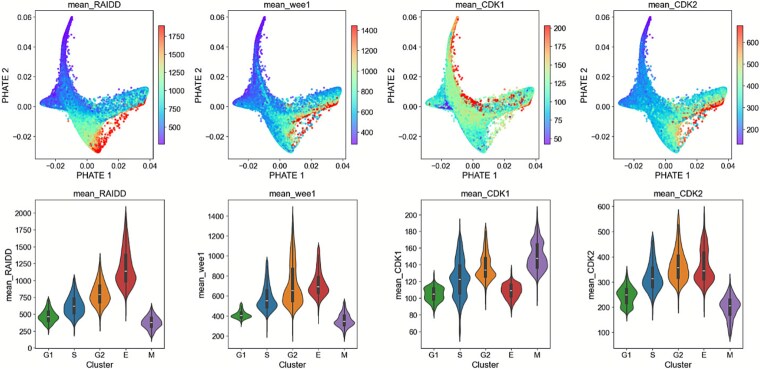
Key proteins governing the bifurcation between endocycling and mitosis. Warmer colors correspond to higher levels of protein expression, whereas cooler colors correspond to lower levels of protein expression. The pathological endocycle is triggered as a stress response and is characterized by a Wee1-mediated inhibition of CDK1 and a switch to CDK2. As the stress is resolved, RAIDD is decreased, and cells can re-enter the cell cycle in G_1_.

Next, we overlaid protein expression levels for RAIDD, Wee1, CDK1, and CDK2, four features that were among the most important to distinguish endocycle cells (as identified by our random forest classifier, differential protein expression across phases, and cell cycle map interrogation), on top of our cell cycle maps (Fig. 4). We note that expression of RAIDD, a component of the PIDDosome complex involved in cellular stress signaling and genome surveillance, was markedly upregulated during the endocycle phase. This suggests that RAIDD may function as part of a stress-activated checkpoint that drives endocycling under pathological conditions. As cellular stress is resolved, RAIDD levels decline, coinciding with re-entry into the canonical cell cycle via G_1_ phase.

**Figure 5:**
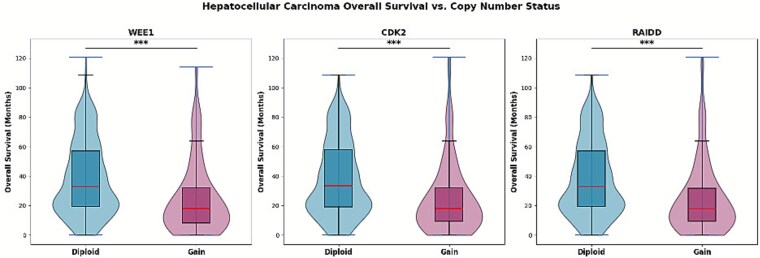
HCC patients with copy number gains in Wee1, CDK2, and RAIDD experience lower overall survival.

We also observed a shift in cyclin-dependent kinase (CDK) activity that reinforces the endocycle phenotype. CDK1, the key driver of mitotic entry, was significantly downregulated during endocycling, while both Wee1 kinase and CDK2 were upregulated as cells entered the endocycle. Wee1 acts to suppress CDK1 activity via inhibitory phosphorylation, thereby preventing mitotic entry and supporting the transition into endoreplication cycles. These findings are supported by a recent study [23] that found that *CDK1^Liv−/−^* mice impaired hepatocyte division, promoted polyploidy, and led to the development of inflammation and fibrosis, even in the absence of any treatment to promote liver damage [23]. Furthermore, concurrent loss of CDK2 reversed polyploidy and the corresponding inflammatory and fibrotic phenotype. Cell cycle maps with protein expression levels for the entire feature set can be seen in Supplemental Fig. 2.

To assess whether high levels of Wee1, CDK2, and RAIDD correlate with poorer clinical outcomes in patients with HCC, we analyzed publicly available data via cBioPortal for Cancer Genomics. Copy number status for Wee1, CDK2, and RAIDD, and overall survival data were obtained for patients across several cohorts, including TCGA and other published studies (*n* = 593, 598, and 594, respectively). Patients were stratified by copy number status (diploid vs. gain) for each gene, and overall survival differences were evaluated using Welch’s *t*-test. We found that gain of Wee1, CDK2, and RAIDD was significantly associated with lower overall survival in HCC patients compared to diploid status (Fig. 5), suggesting that these endocycle-mediating molecular factors may contribute to more aggressive pathology and poorer patient outcomes.

## CONCLUSIONS AND IMPLICATIONS

LHETs are a class of therapeutic strategies that leverage the life history of cells or organisms for improved outcomes. Inspired by ideas from conservation biology and recovery ecology, LHETs have been co-opted in biomedicine for the management of infectious diseases with complex transmission structures. For instance, studying the complex life history of schistosomiasis, which involves parasitic worms, freshwater snails, and waterborne transmission to humans, led researchers to more effective control strategies focused on targeting the snail intermediate for reduced transmission [48–50].

In recent years, we have developed LHETs to combat cancer therapeutic resistance, with promising results [8]. Notably, these studies demonstrate that ignoring the cellular life history of cancer and treating distinct cell states as separate species leads to inaccurate predictions of therapeutic efficacy and suboptimal choice of treatment strategy [5–7, 34, 51]. Conversely, we have recently shown that cell type interactions between the adrenergic and mesenchymal phenotypes in neuroblastoma drive therapy response much more than cell state transitions. This suggests that neuroblastoma may be particularly well-suited for adaptive therapies. Collectively, these findings highlight the importance of understanding and characterizing cellular life histories to design more effective and durable treatments.

In this work, we expanded the scope of LHETs to the problem of cancer prevention, specifically HCC. By reshaping the hepatic microenvironment through senescent, inflammatory, and fibrotic phenotypes that promote cancer invasion, we conceptualize endocycling hepatocytes as a pathological keystone state within the MASH liver microenvironment, generating a population of cells with a disproportionate impact on ecosystem structure and function. This ecosystem engineering effect often manifests as field cancerization with polyclonal HCC origins. Indeed, recent genomic analyses reveal that MASH livers exhibit multiple independent clonal populations simultaneously evolving across cirrhotic nodule [11, 52] and HCCs with multinodular morphology are typically polyclonal with extensive heterogeneity [53]. The endocycle-induced SIF profile creates a mutagenic, cancer-promoting field effect across the liver, with even histologically normal tumor-surrounding sections displaying activated oncogenic pathways [54, 55].

To explore this idea further, we used mathematical modeling to demonstrate how blocking this pathological keystone life history transition can prevent HCC initiation. We then sought to identify appropriate drug targets for this purpose. However, methods to visualize and interrogate life history at a cellular level in fixed diseased tissues were lacking. To this end, we proposed a novel technique called cell cycle mapping, which combines multiplexed immunofluorescence imaging *in situ* with manifold learning to generate visual representations of the life histories of cells from one cell division to the next. Importantly, this is the first method that enables the examination of cellular life histories using fixed tissue samples. Applying this method to a TMA of patients with MASH, we were successfully able to distinguish the endocycle from the canonical cell cycle and identify key proteins that mediate this cell state transition. Our proposed LHETs that target the resulting pathological keystone population to prevent ecosystem degradation fundamentally take a restoration ecology approach that operates at the field-level. Namely, we propose that by blocking endocycling, we may restore the degraded liver ecosystem and prevent the establishment of cascading field effects. As suggested by our cancer, criminology, and corruption research program [45–47], re-establishing the underlying social system (liver microenvironment) may prove to be a more effective and durable solution than mass incarceration of bad agents (killing of individual cancer clones) in the society (tissue).

This data-driven approach to visualizing the life history of cells is a powerful hypothesis-generation tool that can be used to facilitate LHETs. For example, from our analysis of our cell cycle maps, we hypothesize that inhibiting RAIDD, Wee1, and CDK2 will reduce rates of endocycling, and divert cells toward apoptosis via mitotic catastrophe. Critically, this life history approach has significant benefits over traditional therapeutic approaches: Instead of directly targeting endocycled hepatocytes or cancer cells for extinction, an approach that has proven to be rather difficult thus far, we can target *transitions* of cells into the pathological endocycle state and prevent these cells from arising altogether. Analysis of publicly available datasets suggests that these molecular factors are associated with poorer survival outcomes in patients with HCC.

In future work, we will validate these targets using *in vivo* models of MASH-induced HCC with the goal of translating these results into the clinic to hamper the progression of MASH and prevent HCC initiation. To do this, we will use a recently developed mouse model that mimics pathologic sequelae of MASH-induced HCC in humans [56–60] and has been used for testing novel antifibrotic [61] and anti-MASH drugs [62]. We will administer Streptozotocin (100 mg/kg intraperitoneally) to healthy B6 mice at day 2, followed by maintenance on a high-fat diet at week 4. We will then administer the endocycle-targeting therapies identified earlier to the mice using a lipid nanoparticle delivery system [63–65] when they are diagnosed with MASH, occurring at ~8 weeks of age. Mice will be monitored, and HCC formation will be compared to mice that did not receive the endocycle-targeting therapy (grossly visible hepatic tumors form ~25 weeks). In addition to evaluating the number of HCCs formed and time to tumor formation, we will obtain tissue samples for the mice and stain for markers of SIF to assess whether our LHET that targets pathological endocycling can restore liver ecosystem function.

Although we specifically focused on MASH here, our cell cycle mapping approach has much broader implications in the context of LHETs. Pathological cell state transitions occur across biomedicine, from cancer [8, 66–77] to heart disease [78] to kidney disease [18, 20, 79]. In each of these contexts, LHETs that target these alternate cell cycle trajectories may be promising therapeutic avenues. The cell cycle mapping technique proposed here may provide systematic, data-driven ways to visualize and interrogate the cell cycle, informing drug targets for preclinical studies, and helping bring LHETs closer to the clinic.

## Supplementary Material

Supp1_eoag002

Supp2_eoag002

## Data Availability

The data sets of the current study are available from the corresponding author on reasonable request.
