# Supplementary figures and images for: Life history enlightened therapies: cell cycle mapping to identify molecular targets to prevent hepatocellular carcinoma
Source: Evol Med Public Health. 2026 Jan 14;14(1):1–13. doi: 10.1093/emph/eoag002 (PMC12989716; doi:10.1093/emph/eoag002)

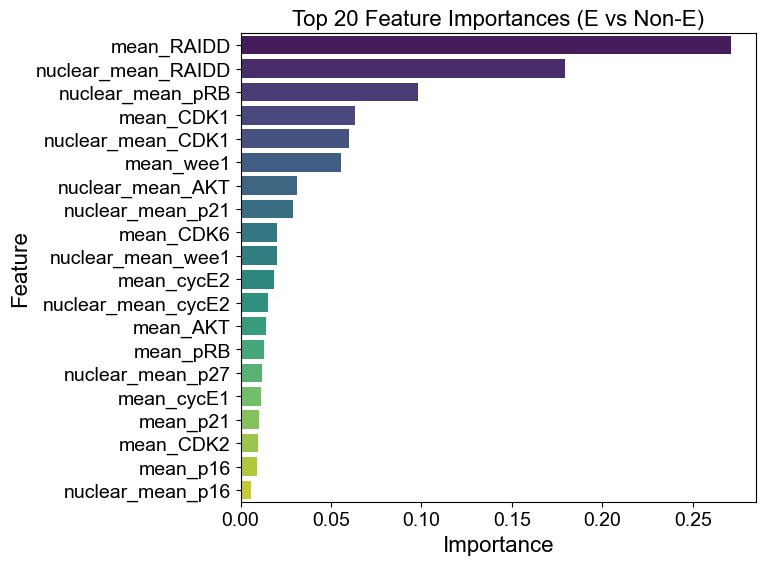

Supplement: Supp1_eoag002 [file supp1_eoag002.jpeg]

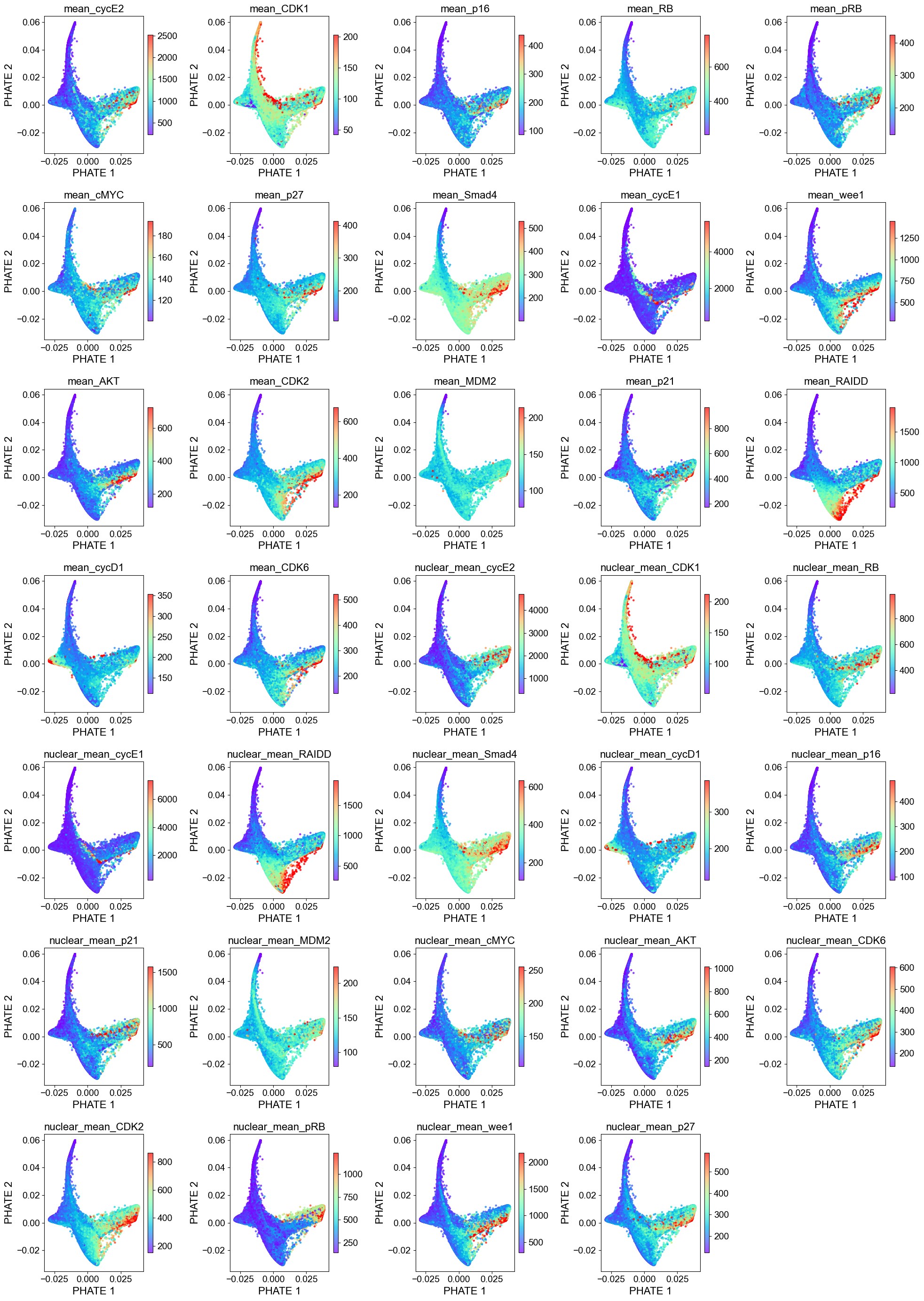

Supplement: Supp2_eoag002 [file supp2_eoag002.jpeg]
